# Transcriptomic analysis: the protection of over-expression thioredoxin reductase 1 in Parkinson’s disease

**DOI:** 10.1186/s41016-023-00319-2

**Published:** 2023-04-03

**Authors:** Zihua Liu, Qiang Ye, Ying Jiang

**Affiliations:** 1 Department of Blood Transfusion Service, the Second Affiliated Hospital of Lanzhou University, Lanzhou, 730030 Gansu Province China; 2 Department of Anatomy and Histology, School of Basic Medical Sciences, Lanzhou University, Lanzhou, China; 3 Intensive Care Center of Gynecology and Obstetrics, Gansu Provincial Maternity and Childcare Hospital, Lanzhou, 730050 Gansu China

**Keywords:** Parkinson's disease, Thioredoxin reductase 1, Na^+^-K^+^-ATP, TLR2, CD14

## Abstract

**Background:**

Parkinson’s disease (PD) is the second most common neurodegenerative disease. The pathologic characteristic feature is the loss of dopaminergic neurons in the substantia nigra (SN). However, the biochemical mechanisms are unclear.

A large number of studies have shown that oxidative damage is the primary cause of PD. Hence, antioxidants could become a suitable option to treat PD. The thioredoxin (Trx) system represents a useful, potentially disease-relevant oxidation–reduction system. Thioredoxin reductase 1 (TR1) is a significant component of the Trx system.

**Methods:**

The overexpression lentivirus (LV) or LV-TR1 in the TR1-A53T model of PD by the stereotactic brain, and successful overexpression of LV or LV-TR1 in the MPP^+^-induced cellular model by LV or LV-TR1 transfection.

**Results:**

We confirmed that interleukin*-7* mRNA levels increased in MPP^+^ compared to that in the control and MPP^+^-TR1 groups using quantitative polymerase chain reaction. The γ-H_2_AX level was increased in the Tg-A53T group compared to that in the TR1-A53T group by western blotting. The expression of Na^+^-K^+^-ATP was decreased in the MPP^+^ group compared to that in the control and MPP^+^-TR1 groups by high content screening. Tg-A53T(the C57BL/6 mice transferred with mutant human a-syn); TR1-A53T(A53T mice which were injected TR1-LV 2 µl in SNc on two sides with minipump).The mice were fed for 10 months. control (the N2a cells cultivated with DMEM); MPP^+^(the N2a cells dealt with MPP^+^(1 mM) 48 h), MPP^+^-LV (the N2a cells over-expressed LV for 24 h then dealt with MPP^+^(1 mM) 48 h). MPP^+^-TR1(the N2a cell over-expressed TR1-LV for 24 h then dealt with MPP^+^(1 mM) 48 h).

From the Kyoto Encyclopedia of Genes and Genomes (KEGG) analysis, we confirmed that the overexpression of TR1 in SN pars compacta cells decreased oxidative stress, apoptosis, DNA damage, and inflammatory response and increased NADPH, Na^+^-K^+^-ATP, and immune response in this PD model.

**Conclusions:**

Our study shows that overexpressed TR1 can be developed as a neuroprotective agent for PD. Therefore, our findings demonstrate a new targeted protein for the treatment of PD.

## Background

Parkinson’s disease (PD) is a neurodegenerative disease. There are more than 6.1 million individuals enduring PD worldwide [1]. Patients with PD experience a major deficit in their quality of life, including their motor [2] and nonmotor symptoms.

PD is a multifactorial disease whose mechanism cannot be confirmed [3]. Multiple factors including genetic mutations [4], neuroinflammation [5], and neuronal excitotoxicity [6] can help explain the reasons underlying the development of PD symptoms. Neuroinflammation and neuronal excitotoxicity illustrate the machinery of an excess of apoptosis and the deficiency of dopaminergic neurons during the progression of PD. The primary clinical symptom of PD is a decrease in dopamine levels in the substantia nigra (SN) and striatum [7]. However, the exact reason for this neurodegeneration remains unknown.

Accumulating evidence suggests that oxidative damage may be involved in several diseases, including tumors, aging, and neurodegenerative diseases [8]. This is because the overproduction of reactive oxygen species (ROS) causes antioxidant systems [9].

The oxidant damage theory has begun to explain the etiology and pathogenesis of PD. Oxidative stress may cause the degeneration of dopaminergic neurons [10] through inadequate removal of ROS or overproduction in cells and tissues, thereby resulting in the oxidant–antioxidant imbalance that induces damage to various cellular components. Moreover, antioxidants may decrease oxidative damage, provide symptomatic relief, and reduce side effects. Recent studies have suggested that excessive oxidative stress is a pivotal factor that contributes to the development and clinical progression of PD [11].

Thioredoxin reductase (TR) has versatile functions including cell growth and differentiation [12]. TR1 plays a critical role in defending against oxidative stress. The brain contains a mass of TR1. To estimate the modification of the disease potential of TR1 in PD, where SN neurons are decreasing and when under stress from exposure to MPTP, we investigated the downregulation of TR1 expression in a mouse model of PD [13]. Thus, we applied a mouse model of PD over-expression of α-syn (A53T) in dopaminergic neurons of the SN, leading to reliable and progressive neurodegeneration [14]. This model is much more similar to the disease process than the toxin models. MPP^+^ is commonly used to generate PD cell models [15]. This study aimed to determine whether TR1 overexpression protects A53T mice and N2a cells from MPP^+^-induced PD both in vitro and in vivo, thus potentially providing a protective enzyme for PD.

## Methods

### Animals

Tg-A53T mice that had overexpressed α-syn were purchased from the Jackson Laboratory and were fed in the animal feeding room of Lanzhou University. The mice were housed in a humidity- and the temperature-controlled environment with a 12/12 light/dark cycle and lights on at 7:30 AM. There were three mice in each group, and the timeline of the study protocol was as follows.

### Cell lines and cell culture

N2a cells were purchased from the Shanghai Cell Research Centre. They were incubated in humidified atmosphere with 5% CO_2_ and 95% O_2_ at 37 °C. The cells were cultured in Dulbecco’s modified Eagle’s medium (DMEM) (Hyclone), which contained 10% fetal bovine serum and penicillin–streptomycin-amphotericin B solution and changed every 2 days.

### Stereotactic injection of lentivirus-TR1 or lentivirus vectors

Tg-A53T mice were anesthetized after feeding for 3 months. A total volume of 2 μl containing 1 × 10^11^ gcs of LV-TR1 vectors was purchased from Shanghai Genechem Co. Ltd. (Cat. No. 00295557) and was injected into the SN. Eye ointment was used to prevent dehydration of the mouse eye. The stereotactic process was performed as described previously [16]. The following coordinates were used: − 4.3 mm ventral, − 3.2 mm lateral, and − 1.2 mm anterior. The wound was sutured. Seven months after LV-TR1 injection, mice were sacrificed by cervical dislocation, and brain tissues were collected for analysis.

### Immunofluorescence

In brief, sections were blotted using rabbit anti-Na^+^-K^+^-ATP at 4 °C overnight. The Na^+^-K^+^-ATP-positive cells were confirmed by monoclonal anti-antibody for 1 h at 37 °C; CY3 (Cat No. RS38111, Immunoway) for 2 h at room temperature. The N2a cells were washed using 0.01 mol/L phosphate-buffered saline, and a high-intensity screening microscope was used to acquire the fluorescence images. Immunofluorescence analysis was performed according to the average fluorescence intensity of each cell.

### RNA sequencing and quantitative polymerase chain reaction (qPCR) analysis

RNA sequencing was performed by OE Biotech Co. Ltd. (Shanghai, China). It was performed according to the method described by Liu et al. [17]. Different genes were determined by *p* value < 0.05 and |Fold Change|> 2.0 or < 0.5.The discovery was according to KEGG pathway enrichment and GO function analyses.

The qPCR analysis was performed according to the method described by Liu et al. [17]. Total RNA from the brain was isolated using the TRizol reagent kit. The concentration of the total RNA was measured by the NanoDrop 2000 spectrophotometer. Reverse transcription was accomplished by PrimeScript RT Master Mix. The Bio-Red system was used to analysis by q-PCR with SYBR premix Ex Taq. Genes primers IL-7 (forward, 5′-TTGCTGCACTGTCATTTGATCC-3′, reverse, 5′-GTCTGCAATCCTAGCCTGCCTTA-3′).The reaction conditions were 95 °C for 10 s, followed by 40 cycles of 95 °C for 5 s and 64 °C for 30 s. The different level was quantified by the 2^−ΔΔCq^ method.

### Western blot analysis

Proteins were denatured in bromophenol blue, separated by sodium dodecyl sulfate–polyacrylamide gel electrophoresis (12% acrylamide) and transferred onto a polyvinylidene fluoride (PVDF) membrane. The membrane was incubated using 5% skim milk powder in Tris-buffered saline with 0.1% Tween 20. The membranes were incubated with primary antibodies against TR1, γ-H_2_AX, or tyrosine hydroxylase (TH) (Abcam). The membranes were incubated using glyceraldehyde 3-phosphate dehydrogenase (Proteintech). The PVDF membranes were then blotted with a secondary antibody, and the bands were tested using contrast-enhanced chemiluminescence.

### Malondialdehyde (MDA), superoxide dismutase (SOD), and glutathione (GSH) production analysis

Lipid peroxidation determination, MDA, SOD, and GSH measurement kits were obtained from Nanjing Jiancheng Bioengineering Institute. The assay was performed as the manufacturer’s instructions.

### Statistical analysis

Data are expressed as mean ± standard error of the mean. Differences between groups were analyzed using SPSS Statistics 26.0 software (IBM, Armonk, NY, USA) by Student’s *t* test. Statistical significance was set at *P* < 0.05. GraphPad Prism 9.0 (GraphPad Software, San Diego, CA, USA) is used to draw the column chart, AI 2019 software (Adobe, Los Altos, CA, USA) was used to design the figures.

## Results

**Fig. 1:**
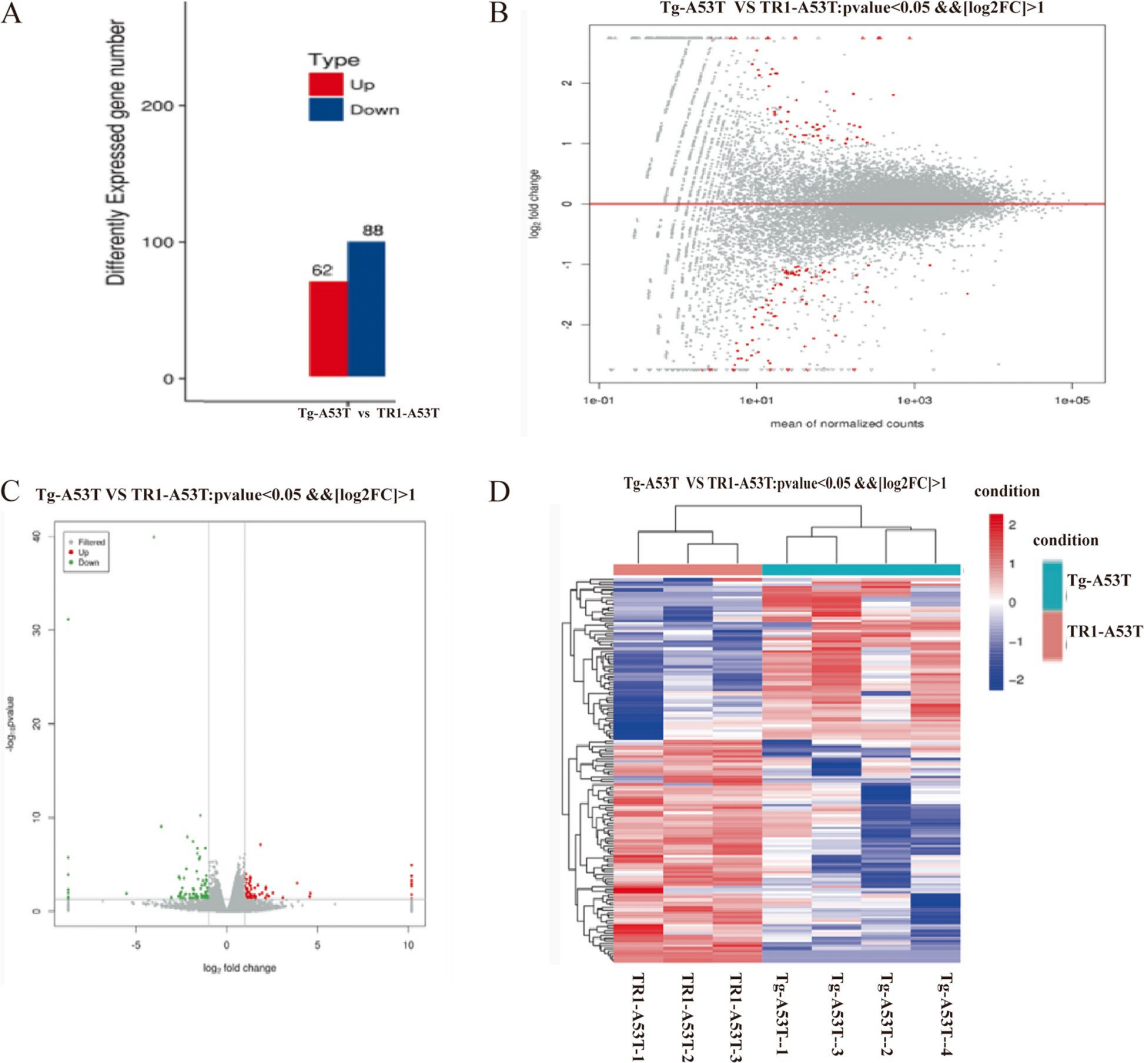
mRNA expression profiles in the Tg-A53T and TR1-A53T groups. **A** Upregulation and downregulation of mRNAs in the Tg-A53T group compared to the TR1-A53T group. **B**, **C** Scatter plot illustrating mRNA expression profiles in Tg-A53T and TR1-A53T. Red represents upregulated genes and green represents downregulated genes. Black represents genes with no significant difference in expression. **D** Heat map and hierarchical clustering show the global expression of mRNA in Tg-A53T compared to the TR1-A53T group. Red and blue indicate high and low relative expressions, respectively. Tg-A53T (C57BL/6 mice transferred with mutant human a-syn); TR1-A53T (A53T mice injected with lentivirus-TR1 2 µl in substantia nigra pars compacta (SNc) on each side with minipump). Mice are fed for 10 months

The mRNA expression profiles in RNA-seq suggested differential expression of 150 mRNAs between the Tg-A53T and TR1-A53T groups, of which the expression levels of 62 mRNAs were increased and those of 88 mRNAs were decreased (Fig. 1A). The heat map shows the expression profiles of all mRNAs (Fig. 1D). The volcano plot indicates mRNAs that were significantly expressed between the two groups (Fig. 1C).

**Fig. 2:**
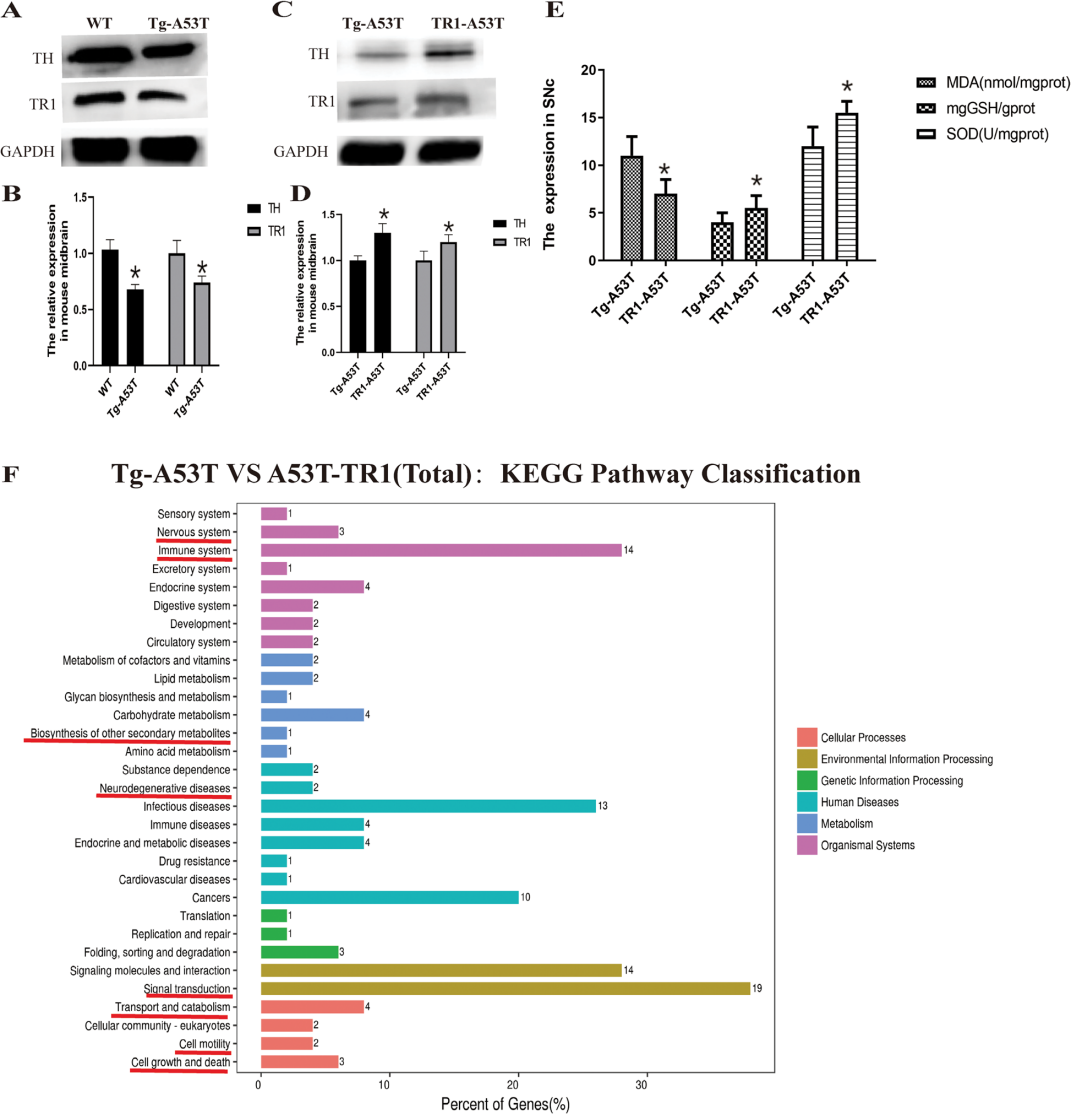
Expression of TR1 and tyrosine hydroxylase (TH), oxidative damage, and total (up and down) differentially expressed mRNAs in Tg-A53T mice compared to TR1-A53T mice. **A** The expression of TR1 and TH is examined in the midbrain of the WT and Tg-A53T groups. **B** Relative expression of TR1 and TH. **C** The expression of TR1 and TH is examined in the midbrain in the Tg-A53T and TR1-A53T groups. **D** Relative expression of TR1 and TH. *p < 0.05, Tg-A53T *vs.* TR1-A53T group. **E** GSH, SOD, and MDA levels. **F** The bar indicates the* p* value (–log-transformed) the number of changed mRNAs in each pathway. The *p* value (Fisher *p* value) denotes the significance of the pathway correlated to the conditions. A lower *p* value (*p* value ≤ 0.05, considered statistically significant) denotes a more significant correlation

The expression of TR1 and TH in the midbrain of Tg-A53T was lower in the Tg-A53T group than in the WT group. Additionally, the expression of TR1 and TH significantly increased in the midbrain of TR1-A53T mice that in Tg-A53T mice. There were increased levels of GSH and SOD in the TR1-A53T group than those in the Tg-A53T group and decreased MDA expression in TR1-A53T than in the Tg-A53T group. The Kyoto Encyclopedia of Genes and Genomes (KEGG) pathway analysis suggested 31 substantially enriched pathways, many of which are relevant to the nervous system, immune system, biosynthesis of other metabolic pathways, neurodegenerative diseases, signal transduction, transport and catabolism, cell motility, cell growth, and death in the Tg-A53T group compared with those in the TR1-A53T group Fig. 2.

**Fig. 3:**
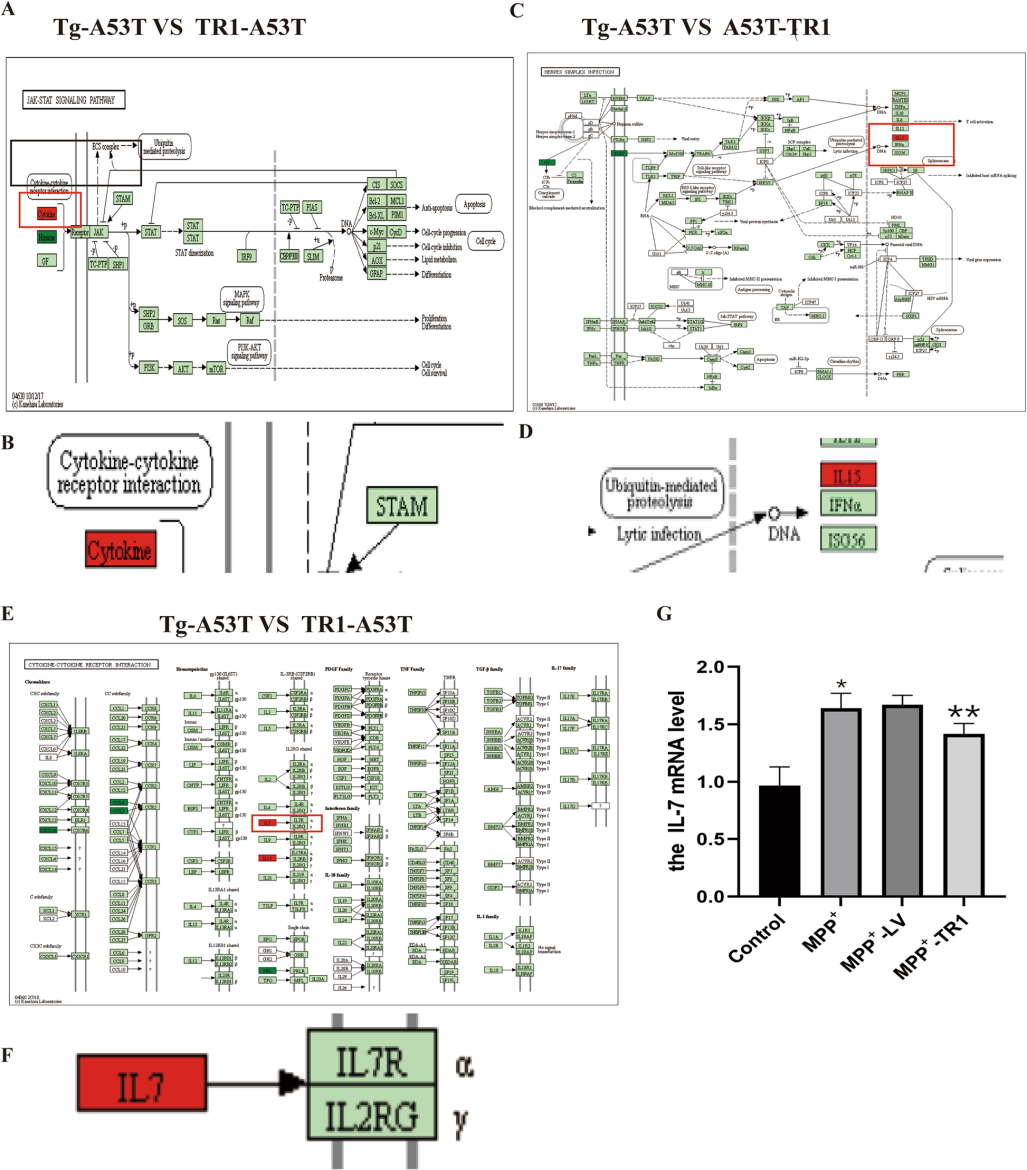
Overexpression of TR1 decreases the expression of* interleukin (IL)-7*, *IL-15*, and *cytokines* in Tg-A53T than that in the TR1-A53T group. **A**, **B** KEGG pathway analysis of cytokine expression in the Tg-A53T and TR1-A53T groups. **B** Enlargement in **A**. **C**, **D** KEGG pathway analysis the expression of interleukin (*IL)-15* in Tg-A53T and TR1-A53T groups. **D** Enlargement in **C**. **E**, **F** KEGG pathway analysis of the expression of *IL-7* in the Tg-A53T and TR1-A53T groups. **F** The enlargement in **E**. **G** mRNA expression of *IL-7* in control, MPP^+^, MPP^+^-LV, and MPP^+^-TR1 groups. Control (N2a cells cultivated with DMEM); MPP^+^ (N2a cells used MPP^+^(1 mM) for 48 h), MPP^+^-LV (N2a cells overexpressing lentivirus for 24 h then used MPP^+^ (1 mM) for 48 h. MPP^+^-TR1 (N2a cells overexpressing lentivirus-TR1 for 24 h, followed by MPP.^+^ (1 mM) for 48 h

KEGG pathway analysis showed that the expression of *cytokines*, *IL-15*, and *IL-7* was upregulated in the Tg-A53T group compared with that in the TR1-A53T group. The mRNA expression of *IL-7* was upregulated in MPP^+^ compared with that in MPP^+^-TR1 in the control, MPP^+^, MPP^+^-LV, and MPP^+^-TR1 groups Fig. 3.

**Fig. 4:**
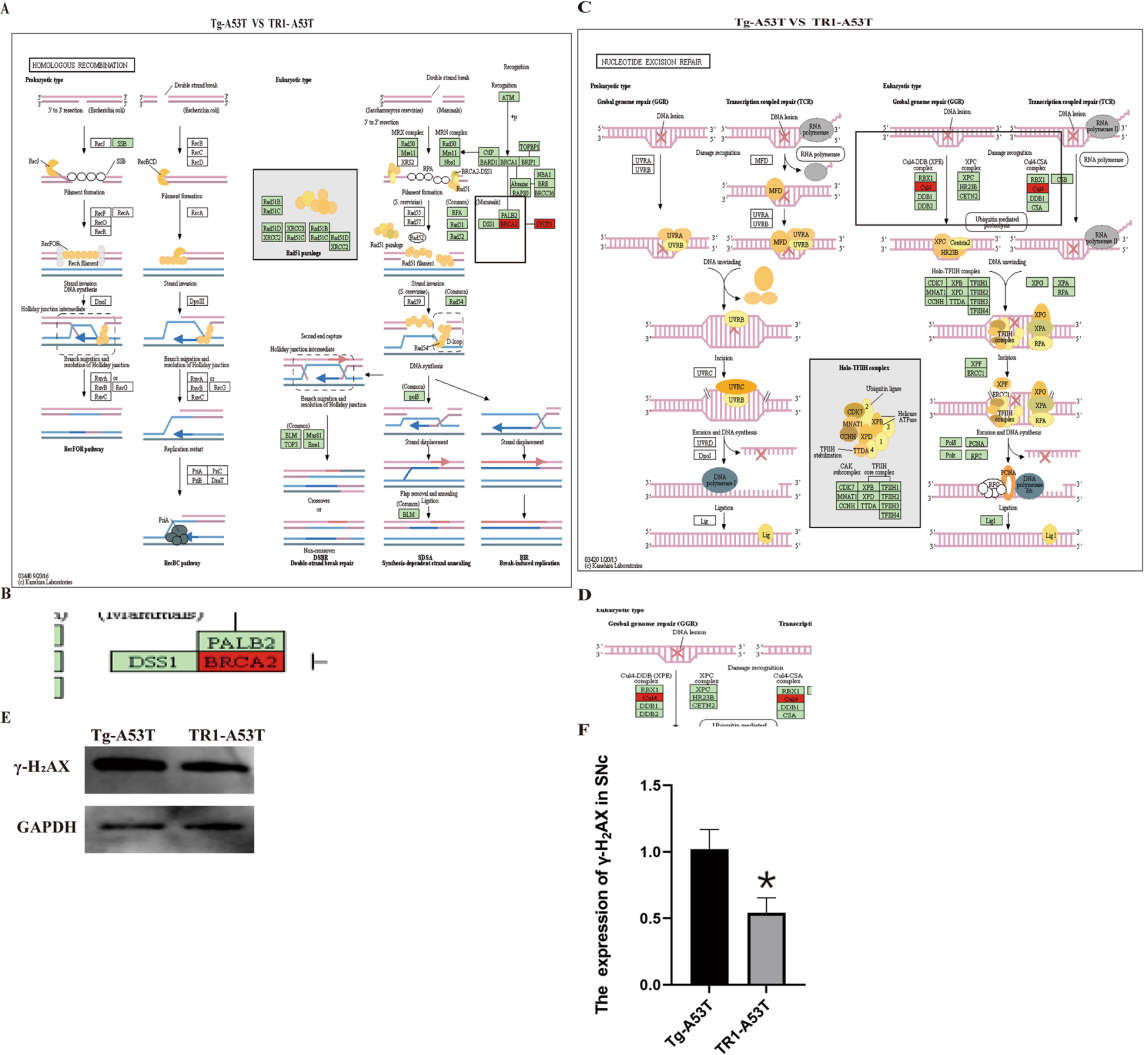
Overexpression of TR1 decreases the expression of *BRCA2*, *CUL4*, and γ-H_2_AX in the Tg-A53T group compared to the TR1-A53T group. **A**, **B** KEGG pathway analysis of the expression of *BRCA2* in the Tg-A53T and TR1-A53T groups. **B** Enlargement in **A**. **C**, **D** KEGG pathway analysis of the expression of *CUL4* in the Tg-A53T and TR1-A53T groups. **D** The part of enlargement in **C**. **E**, **F** The expression of γ-H_2_AX in the SNc in the midbrain of the mice. *p < 0.05, Tg-A53T mice *vs.* TR1-A53T mice

KEGG pathway analysis showed that the expression of *BRCA2 and CUL4* was upregulated in the Tg-A53T compared to the TR1-A53T; the expression of γ-H_2_AX was upregulated in Tg-A53T compared with the TR1-A53T group Fig. 4.

**Fig. 5:**
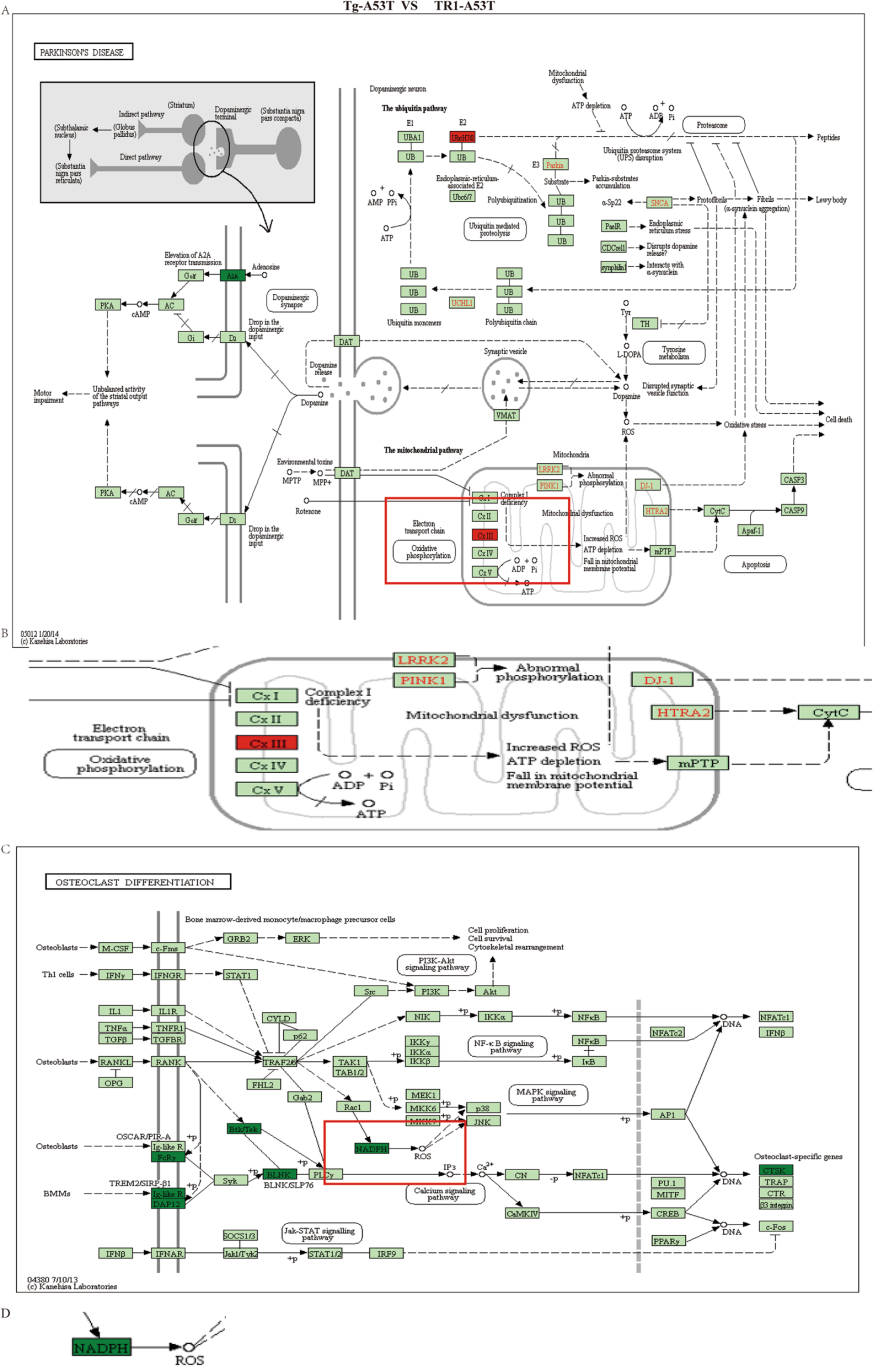
Overexpression of TR1 increases the expression of *NADPH* and decrease* cytochrome b (cxIII)* in Tg-A53T compared with the TR1-A53T group. **A**, **B** KEGG pathway analysis of the expression of *cxIII* in the Tg-A53T and TR1-A53T groups. **B** Enlargement in **A**. **C**, **D** KEGG pathway analysis of the expression of *NADPH* in the Tg-A53T and TR1-A53T groups. **D** Part of the enlargement in **C**

KEGG pathway analysis showed that the expression of *cxIII* was upregulated in Tg-A53T compared with the TR1-A53T group, while the expression of *NADPH* was downregulated in Tg-A53T compared with that in the TR1-A53T group Fig. 5.

**Fig. 6:**
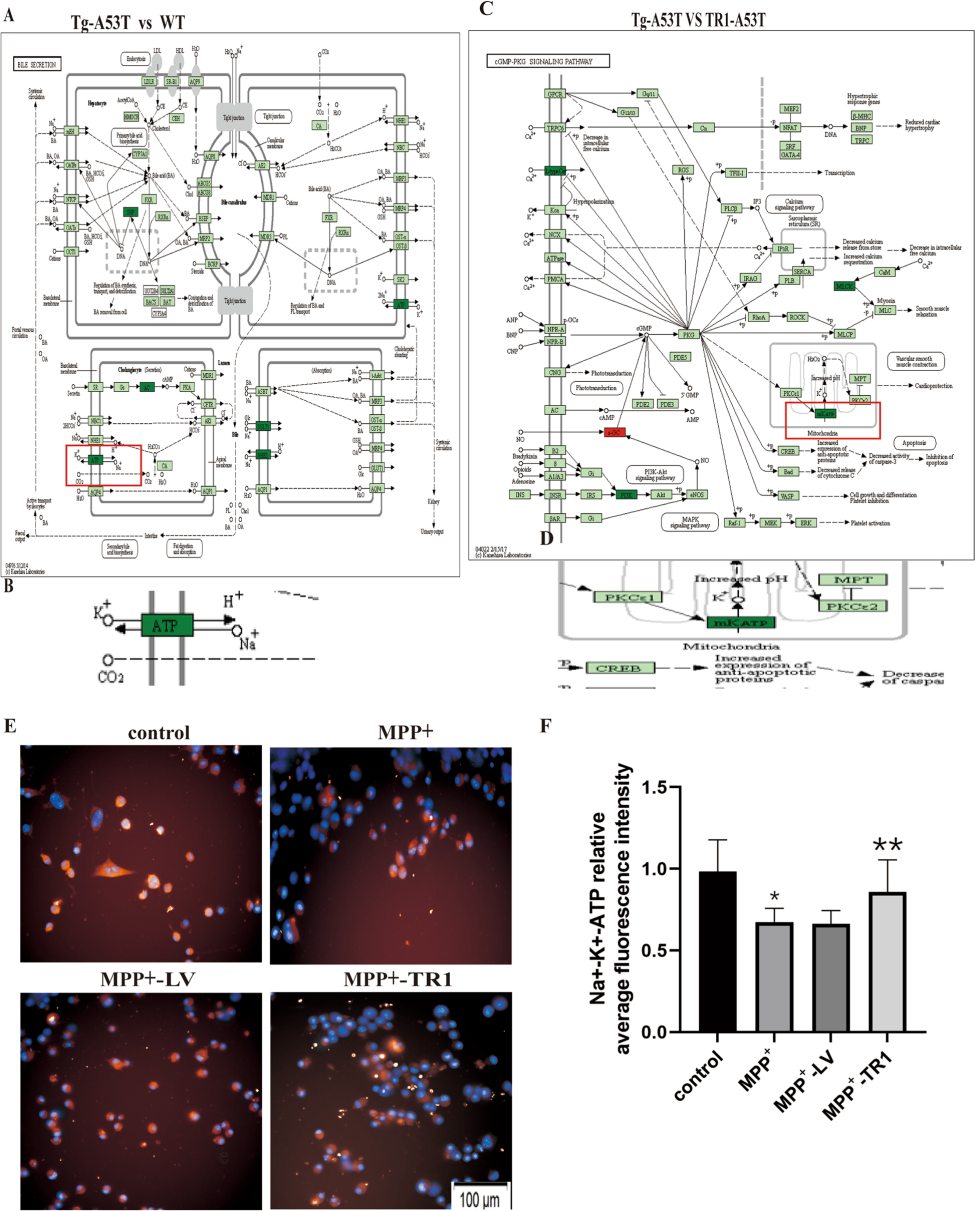
Over-expression of TR1 increases the expression of Na^+^-K^+^-ATP in PD mouse and cell models. **A** KEGG pathway analysis of the expression of *Na*^+^*-K*^+^*-ATP* in the Tg-A53T and WT groups. **B** Enlargement in **A**. **C** KEGG pathway analysis of the expression of *Na*^+^*-K*^+^*-ATP* in the Tg-A53T and TR1-A53T groups. **D** The part of enlargement in **C**. **E** The expression of Na^+^-K^+^-ATP in control, MPP^+^, MPP^+^-LV, and MPP^+^-TR1 groups by immunofluorescence. **F** Relative average fluorescence intensity of Na^+^-K^+^-ATP. *p < 0.05, MPP^+^*vs.* control group, **p < 0.05, MPP^+^*vs.* MPP^+^-TR1 group. WT (normal C57BL/6 mice). Control (N2a cells cultivated with DMEM); MPP^+^(N2a cells used MPP^+^(1 mM) for 48 h), MPP^+^-LV (N2a cells overexpressing lentivirus for 24 h then used MPP^+^ (1 mM) for 48 h. MPP^+^-TR1(N2a cells overexpressing lentivirus-TR1 for 24 h, followed by MPP.^+^(1 mM) for 48 h)

KEGG pathway analysis showed that the mRNA level of Na^+^-K^+^-ATP was down-regulated in the Tg-A53T compared to that of the WT group, and the mRNA level of Na^+^-K^+^-ATP was down-regulated in the Tg-A53T compared to that of the TR1-A53T group; the expression of Na^+^-K^+^-ATP decreases in the MPP^+^ compared with that of the control (*p < 0.05); the expression of Na^+^-K^+^-ATP decreases in the MPP^+^ compared with MPP^+^-TR1 (**p < 0.05) in the N2a cells that were separated from the control, MPP^+^, MPP^+^-LV, and MPP^+^-TR1 groups Fig. 6.

**Fig. 7:**
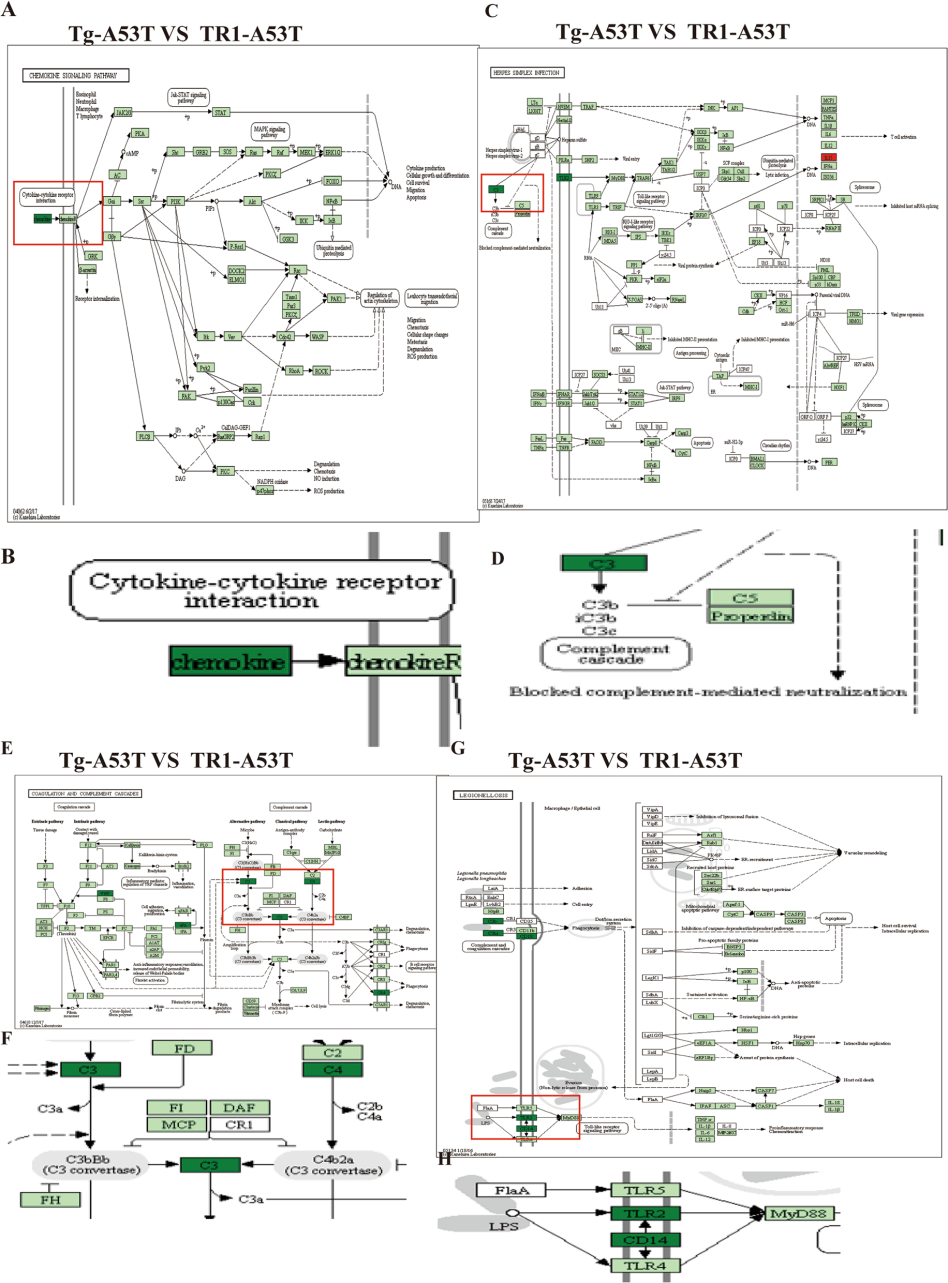
Over-expressed TR1 can increase the expression of** chemokine**, *C3, C4, TLR2, CD14 *in the Tg-A53T mice compared to TR1-A53T mice. **A** KEGG pathway analysis of chemokine expression in the Tg-A53T and thioredoxin reductase 1 (TR1)-A53T groups. **B** Enlargement in **A**. **C** KEGG pathway analysis of the expression of *C3* in the Tg-A53T and TR1-A53T groups. **D** The part of enlargement in **C**. **E** KEGG pathway analysis the expression of *C4* in Tg-A53T and TR1-A53T groups. **F** Enlargement in **E**. **G** KEGG pathway analysis of the expression of *TLR2 *and* CD14* in the Tg-A53T and TR1-A53T groups. **F** Part of the enlargement in **E**

KEGG pathway analysis showed that the expression of *chemokine*, *C3,* and *C4* was down-regulated in the Tg-A53T compared to that of the TR1-A53T group. KEGG pathway analysis suggested that the expression of *TLR2* and *CD14* was downregulated in the Tg-A53T compared to that of the TR1-A53T group Fig. 7.

## Discussion

We can deduct from the KEGG pathway analysis that antioxidative activity has a critical role in the TR1-A53T group compared with that in the Tg-A53T group. To counteract the redox stress, SN pars compacta (SNc) cells have to boost their antioxidant capacity exhaustively to maintain a sensitive redox balance in the PD model.

We demonstrated that the overexpression of TR1 can increase *NADPH* expression levels and prevent an increase in ROS. ROS can produce many mitochondria-associated events such as apoptosis [18]. However, antioxidant enzymes, including TR, can disrupt cellular ROS homeostasis and defense against oxidative damage, thereby regulating many biological processes, including cell differentiation, death, and proliferation [19]. In contrast, ROS can damage proteins, DNA, and lipids, resulting in cell death by causing a series of redox imbalances. To counteract the redox stress, SNc cells have to boost their antioxidant capacity to maintain a sensitive redox balance in the PD model.

DNA damage is in advance of the degeneration of dopamine neurons in Tg-A53T mice [20] in our previous study. In this study, we suggested that overexpression of TR1 in the PD model decreases the levels of *BRCA2*, *CUL4*, and γ-H_2_AX compared with that of Tg-A53T; therefore, TR1 can protect against the DNA damage as shown in the vivo and in vitro experiments in the PD model (Fig. 4).

This study revealed that improving the level of TR1 may inhibit dopaminergic neuronal death. Moreover, overexpression of TR1 protects Tg-A53T against synaptic loss induced by TH, the mechanisms driving the switch during PD progression and their correlation remain to be elucidated.

Our study suggests that overexpression of TR1 in Tg-A53T can reduce inflammation. Neuroinflammation has been repeatedly linked to neurodegeneration, including PD, and inflammation can enhance α-syn aggregation, propagation, and progression of the disease [21]. Increased activation of the production of pro-inflammatory cytokines alters the neuronal function and increases cell death in models of PD and other neurodegenerative diseases [22, 23]. IL-1β and IL-6 result in neuronal dysfunction in PD [24]. Cytokines are known to trigger microglial activation, potentially contributing to nigrostriatal pathway injury. As dopaminergic neurons express a wide range of cytokine receptors, they are suggested to be responsive to these inflammatory mediators, which are derived from or activate microglia. This confirms the involvement of microglia in the initiation of anti-inflammatory events, pointing toward the existence of multiple phenotypes in PD and distinct functions during disease progression. Cytokines may diffuse easily from the brain through the blood–brain barrier [25]. In this study, we showed that overexpression of TR1 in Tg-A53T in the midbrain, the expression of *IL-7*, cytokines*,* and *IL-15* is downregulated in TR1-A53T compared with Tg-A53T. Therefore, overexpression of TR1 in the SN of the midbrain can inhibit inflammation in Tg-A53T, the reason for which is unknown.

Our study suggests that overexpression of TR1 in Tg-A53T cells can increase the immune response. *C3* is the central complement system protein and is involved in both classical and alternative pathway cascades. *C4*, in addition to *C3*, is a marker of the classical complement pathway. Both *C3* and *C4* are acute-phase proteins. Complement-mediated neuroinflammation has also been reported in neurodegenerative diseases [26]. Targeting the immune system is a promising strategy for the treatment of PD. The immunization of WT using α-syn can reportedly decrease protein accumulation in neuronal cell bodies, reduce neurodegeneration [27], and increase CD4^+^ T cells and microglial activation [28].

Toll-like receptors (TLRs) play a critical role in innate immunity by recognizing conserved motifs primarily found in microorganisms, and dysregulation of their signaling may be implicated in α-syn dysfunction. As detected in PD, TLR, possibly exerting proinflammatory effects under certain conditions, triggers downstream signaling pathways, promoting inflammation and oxidative stress in patients with PD. Thus, the gut microbiota and TLRs represent potential targets for PD treatment. In contrast, TLR2 and TLR4 might be essential for clearing misfolded α-syn; hence, being neuroprotective [29]. TLR2 and TLR4 are the most convincing evidence of PD. We found that overexpression of TR1 in the Tg-A53T cells in the midbrain, the expression of TLR2 was upregulated in the TR1-A53T than that in the Tg-A53T group. Therefore, overexpression of TR1 can increase immunology. From Chupakhin E et al. [30] study, we know that TR can defense against oxidative stress and consider an anti-cancer drug target, anti-inflammation, and neonatal hyperoxic lung injury. The mechanism of TR1 improving immunity will be further studied in the future.

## Conclusion

In summary, this study demonstrates the neuroprotective actions of over-expression TR1 in inflammation, the immune response, Na^+^-K^+^-ATP, DNA damage, and in cell apoptosis in Tg-A53T by the KEGG signal pathway. The current findings may suggest a novel paradigm to study the pathology of PD and to provide a new disease-modifying therapeutic approach.

## Data Availability

If requested, the authors agree to provide the Editor copies of the original data.

## Abbreviations

PD: Parkinson’s disease
SN: Substantia nigra (SN)
TR: Thioredoxin reductase
DMEM: Dulbecco’s modified Eagle’s medium
MDA: Malondialdehyde
qPCR: Quantitative polymerase chain reaction
SOD: Superoxide dismutase
GSH: Glutathione

## References

[1] Collaborators, G. B. D. P. s. D (2018). Global, regional, and national burden of Parkinson's disease, 1990-2016: a systematic analysis for the Global Burden of Disease Study 2016. Lancet Neurology.

[2] Titova N, Padmakumar C, Lewis SJ, Chaudhuri KR (2017). Parkinson’s: a syndrome rather than a disease?. J Neural Transm (Vienna).

[3] Silva B, Breydo L, Fink A, Uversky VN (2013). Agrochemicals, α-synuclein, and Parkinson’s disease. Molec Neurobiol.

[4] Cao M, Wu Y, Ashrafi G, McCartney AJ, Wheeler H, Bushong EA, Boassa D, Ellisman MH, Ryan TA, De Camilli P (2017). Parkinson sac domain mutation in synaptojanin 1 impairs clathrin uncoating at synapses and triggers dystrophic changes in dopaminergic axons. Neuron.

[5] Hickman S, Izzy S, Sen P, Morsett L, El Khoury J (2018). Microglia in neurodegeneration. Nat Neurosci.

[6] Litim N, Morissette M, Di Paolo T (2017). Metabotropic glutamate receptors as therapeutic targets in Parkinson's disease: an update from the last 5 years of research. Neuropharmacology.

[7] Petzinger G, Holschneider D, Fisher B (2015). The effects of exercise on dopamine neurotransmission in Parkinson’s disease: targeting neuroplasticity to modulate basal ganglia circuitry. Brain Plast.

[8] Briones AM, Touyz RM (2010). Oxidative stress and hypertension: current concepts. Curr Hypertens Rep.

[9] Fukai T, Ushio-Fukai M (2011). Superoxide dismutases: role in redox signaling, vascular function, and diseases. Antioxid Redox Signal.

[10] Singh R, Barden A, Mori T (2001). Beilin L., Advanced glycation end-products: a review. Diabetologia.

[11] Hwang O (2013). Role of oxidative stress in Parkinson's disease. Experimental Neurobiology.

[12] Nordberg J, Arnér ESJ (2001). Reactive oxygen species, antioxidants, and the mammalian thioredoxin system. Free Radical Biol Med.

[13] Zihua Liu, Yuhong Jing, Jie Yin, Jiying Mu, Tingting Yao, and Liping Gao, Ph.D. Downregulation of thioredoxin reductase 1 expression in the substantia nigra pars compacta of Parkinson's disease mice. Neural Regen Res. 2013;8(35): 3275–3283. 10.3969/j.issn.1673-5374.2013.35.002 PMC4145943 25206649

[14] Koprich JB, Johnston TH, Huot P (2011). Progressive neurodegeneration or endogenous compensation in an animal model of Parkinson’s disease produced by decreasing doses of alphasynuclein. PLoS ONE.

[15] Tetrud JW, Langston JW (1989). MPTP-induced parkinsonism as a model for Parkinson’s disease. Acta Neurol Scand Suppl.

[16] Salinas Tejedor L, Berner G (2015). Mesenchymal stem cells do not exert direct beneficial effects on CNS remyelination in the absence of the peripheral immune system. Brain Behav Immun.

[17] Liu Z, Ye Q, Wang F, Guo Y, Cui R, Wang J, Wang D. Overexpression of thioredoxin reductase 1 can reduce DNA damage, mitochondrial autophagy and endoplasmic reticulum stress in Parkinson's disease. Exp Brain Res. 2020. 10.1007/s00221-020-05979-5 33230666

[18] Mahlknecht P, Gasperi A, Djamshidian A, Kiechl S, Stockner H, Willeit P (2018). Performance of the Movement Disorders Society criteria for prodromal Parkinson’s disease: a population-based 10-year study. Mov Disord.

[19] Koc A, Mathews CK, Wheeler LJ, Gross MK, Merrill GF (2006). Thioredoxin is required for deoxyribonucleotide pool maintenance during S phase. J Biol Chem.

[20] Stafford WC, Peng X, Olofsson MH (2018). Irreversible inhibition of cytosolic thioredoxin reductase 1 as a mechanistic basis for anticancer therapy. Sci Transl Med.

[21] Yoshioka J, Schreiter ER, Lee RT (2006). Role of thioredoxin in cell growth through interactions with signaling molecules. Antioxid Redox Signal.

[22] Powis G, Gasdaska JR, Gasdaska PY (1997). Selenium and the thioredoxin redox system: effects on cell growth and death. Oncol Res.

[23] Ren X, Zou L, Zhang X, Branco V, Wang J, Carvalho C (2017). Redox signaling mediated by thioredoxin and glutathione systems in the central nervous system. Antioxid Redox Signal.

[24] Booze ML, Hansen JM, Vitiello PF (2016). A novel mouse model for the identification of thioredoxin-1 protein interactions. Free Radic Biol Med.

[25] Huang SH, Wu LW, Huang AC, Yu CC (2012). Benzyl isothiocyanate (BITC) induces G2/M phase arrest and apoptosis in human melanoma A375S2 cells through reactive oxygen species (ROS) and both mitochondria-dependent and death receptor-mediated multiple signaling pathways. J Agric Food Chem.

[26] Arnér ESJ, Holmgren A (2006). The thioredoxin system in cancer-introduction to a thematic volume of Seminars in Cancer Biology. Semin Cancer Biol.

[27] Degui Wang, Tianyu Yu, Yongqiang Liu, Jun Yan , Yingli Guo, Yuhong Jing, Xuguang Yang, Yanfeng Song, Yingxia Tian. DNA damage preceding dopamine neuron degeneration in A53T human a-synuclein transgenic mice Biochemical and Biophysical Research Communications. 2016;481:104–110. 10.1016/j.bbrc.2016.11.008 27818201

[28] Bender A, Krishnan KJ, Morris CM, Taylor GA, Reeve AK, Perry RH, Jaros E, Hersheson JS, Betts J, Klopstock T, Taylor RW, Turnbull DM (2006). High of mitochondrial DNA deletions in substantia nigra neurons in aging and Parkinson disease. Nat Genet.

[29] Adamec E, Vonsattel JP, Nixon RA (1999). DNA strand breaks in Alzheimer's disease. Brain Res.

[30] Chupakhin E, Krasavin M (2021). Thioredoxin reductase inhibitors: updated patent review (2017-present). Expert Opin Ther Pat.

